# Noise sensitivity associated with nonrestorative sleep in Chinese adults: a cross-sectional study

**DOI:** 10.1186/s12889-021-10667-2

**Published:** 2021-04-01

**Authors:** Sha Li, Daniel Yee Tak Fong, Janet Yuen Ha Wong, Bradley McPherson, Esther Yuet Ying Lau, Lixi Huang, Mary Sau Man Ip

**Affiliations:** 1 School of Nursing, Li Ka Shing Faculty of Medicine, The University of Hong Kong, 21 Sassoon Road, Pokfulam, Hong Kong, China; 2 Division of Speech and Hearing Sciences, Faculty of Education, The University of Hong Kong, Pokfulam, Hong Kong, China; 3 Sleep Laboratory, Department of Psychology, The Education University of Hong Kong, 10 Lo Ping Road, Tai Po, New Territories Hong Kong, China; 4 Centre for Psychosocial Health, The Education University of Hong Kong, 10 Lo Ping Road, Tai Po, New Territories Hong Kong, China; 5 Department of Mechanical Engineering, The University of Hong Kong, Pokfulam, Hong Kong, China; 6 Department of Medicine, Li Ka Shing Faculty of Medicine, The University of Hong Kong, 21 Sassoon Road, Pokfulam, Hong Kong, China

**Keywords:** Actigraphy, Noise, Noise sensitivity, Nonrestorative sleep

## Abstract

**Background:**

Nonrestorative sleep is a common sleep disorder with a prevalence ranging from 1.4 to 35%, and is associated with various psychological and physical health issues. Noise exposure and noise sensitivity have been proposed to contribute to nonrestorative sleep. This study aimed to examine the relationships among noise, noise sensitivity, nonrestorative sleep, and physiological sleep parameters in Chinese adults.

**Methods:**

A cross-sectional household survey was conducted with randomly selected Chinese adults based on a frame stratified by geographical districts and types of quarters in Hong Kong. We administered a battery of questionnaires, including the Nonrestorative Sleep Scale, the Weinstein Noise Sensitivity Scale, the ENRICHD Social Support Instrument, the Patient Health Questionnaire, and the Perceived Stress Scale to assess nonrestorative sleep, noise sensitivity, social support, somatic symptoms and stress, respectively. Anxiety and depression were evaluated by the Hospital Anxiety and Depression Scale while sociodemographic and lifestyle characteristics were assessed with an investigator-developed sheet. Nocturnal noise level and physiological sleep parameters were measured during nighttime for a week by noise dosimetry and actigraphy, respectively. A structured multiphase linear regression was conducted to estimate associations.

**Results:**

A total of 500 adults (66.4% female) with an average age of 39 years completed this study. Bivariate regressions showed that age, marital status, occupation, family income, season, exercise, cola and soda consumption, social support, somatic symptoms, stress, depression, noise sensitivity, total sleep time, and awakenings were associated with nonrestorative sleep. In the multivariable analysis, family income, season, exercise, social support, somatic symptoms, stress, and depression remained associated with nonrestorative sleep. Specifically, a one-unit increase of noise sensitivity was associated with 0.08 increase in nonrestorative sleep (95% confidence interval [CI]: 0.01, 0.15, *p* = 0.023). Nocturnal noise was negatively associated with time in bed (b = − 1.65, 95% CI: − 2.77, − 0.52, *p* = 0.004), total sleep time (b = − 1.61, 95% CI: − 2.59, − 0.62, *p* = 0.001), and awakenings (b = − 0.16, 95% CI: − 0.30, − 0.03, *p* = 0.018), but was not associated with nonrestorative sleep.

**Conclusions:**

Nonrestorative sleep was predicted by noise sensitivity in addition to family income, season, exercise, social support, somatic symptoms, stress, and depression.

## Background

Nonrestorative sleep refers to the feeling of being unrefreshed and restless upon waking up [1] and was listed as a symptom of primary insomnia [2]. However, nonrestorative sleep can occur without other insomnia symptoms [3]. In the Diagnostic and Statistical Manual of Mental Disorders-Fifth Edition (DSM-V), individuals may be diagnosed as “other specified insomnia disorder” or “unspecified insomnia disorder” when they report nonrestorative sleep without any other sleep disorders [4]. The prevalence of nonrestorative sleep among the general adult population varies from 1.4 to 35% [1]. In Hong Kong, the prevalence and persistence of nonrestorative sleep in adults were reported to be 8 and 31.9%, respectively, which were determined based on a single question about nonrestorative sleep [5]. This unrefreshed feeling may cause fatigue and reduced physical capacity during the day, which may hinder work performance and cause occupational injury [6]. Individuals with nonrestorative sleep also experience reduced psychological well-being and more suicidal ideation [7]. Moreover, several chronic diseases were demonstrated to be highly associated with nonrestorative sleep [8]. Therefore, nonrestorative sleep has gained increasing attention as a potential target of treatment intervention [3]. However, the cause of nonrestorative sleep is still unclear. With reference to the social-ecological model of sleep health, factors from individual, social and societal levels should be considered when identifying the determining factors of sleep problems [9].

Noise pollution is ranked one of the world’s largest environmental problems, second only to air pollution [10]. In Hong Kong, it is one of the most complained about environmental issues [11]. Noise exposure from transportation, construction, community, or social sources has posed a serious burden to humans. The negative health effects induced by noise, such as hearing loss, hypertension, sleep, and cognitive impairment, have been well studied, with a growing number of noise-related additional health problems and diseases [12–15]. For example, it was estimated that more than 30% of the European people were exposed to nocturnal noise levels greater than 55dBA, and 903,000 disability-adjusted life years were lost owing to noise-induced sleep disturbance [12]. The hypothesized link between the physical, auditory stimulus of noise and non-auditory, physiological health effects has been based on the general stress model. Specifically, noise may induce annoyance that results in the release of stress-related cortisol, leading to various adverse physiological and psychological changes [12, 16]. This link also applies to sleep [17]. The release of cortisol can hinder the production of melatonin and then disturb sleep [18]. Interruption of sleep and the adverse physiological and psychological changes may establish a vicious cycle that exacerbates sleep disorders and health problems [19, 20].

Moreover, people who were more sensitive to noise might be more vulnerable to the impact of noise when exposed to the same noise level as others with less sensitivity, reflected by increases in annoyance, and physical and mental health problems. Noise sensitivity is a stable trait that refers to increased reactivity to noise in general [21, 22]. It was also proposed to be associated with emotional responses, especially a tendency to negative feelings of sensations, events and self [22]. In the general population, it was estimated that 20 to 40% of individuals were sensitive to noise, and 12% were highly sensitive to noise [23]. Noise sensitivity might impact health directly or moderate the association between noise and health [24]. A previous study suggested that it was the subjective response to noise or susceptibility to noise, such as noise annoyance or noise sensitivity, respectively, that explained the association of sleep quality complaints after excess noise exposure rather than objective noise itself [25]. Although it has been reported that people who were more tolerant of noise experienced less nonrestorative sleep [26], the mechanism underlying the potential effect of noise sensitivity on nonrestorative sleep remains unclear. It may be that one is more reactive to noise during the night, which is consistent with the stress model for the non-auditory impact of noise. However, one may not experience emotional responses when asleep [27]. A previous study also indicated a much stronger relationship between noise sensitivity and sleep problems than that mediated by annoyance, which was related to the “stress” activity [28]. Therefore, we hypothesized there was a direct association between noise sensitivity and nonrestorative sleep.

In addition to noise, some other factors were proposed to correlate with sleep and stress. For instance, more somatic symptoms, anxiety and depression were associated with a higher risk of nonrestorative sleep as well as a higher stress level [29, 30]. Conversely, exercise and social support were found to be protective factors for sleep problems and to be stress relievers [31–33]. Moreover, lifestyle factors such as consumption of caffeine and diseases such as obstructive sleep apnea might impact nonrestorative sleep through its influences on sleep initiation or sleep maintenance which can also cause stress feelings [34–36]. To conclude, despite the mechanism of nonrestorative sleep is still unclear, we propose that sociodemographic characteristics, lifestyle, noise, noise sensitivity, as well as individual physical and psychological conditions, might act as stressors that cause stress-related physiological activities, and/or act as moderators that impact nonrestorative sleep, with possible direct associations in addition to stress.

To the best of our knowledge, the impact of environmental factors such as noise on nonrestorative sleep, the interaction of noise with personal factors, and the relationship between physiological sleep parameters and nonrestorative sleep still remain to be studied. Furthermore, no study had controlled for factors such as nocturnal noise, stress, depression, and physiological sleep parameters before assessing the association between noise sensitivity and nonrestorative sleep. Moreover, no study used validated scales to assess nonrestorative sleep when examining its association with noise sensitivity, which may have resulted in low measurement sensitivity and reliability. Hence, this study aimed to examine the association of noise, noise sensitivity with nonrestorative sleep and physiological sleep parameters, and the moderating effect of noise sensitivity, among Chinese adults in Hong Kong. We hypothesized that higher noise exposure and noise sensitivity would be associated with higher nonrestorative sleep and physiological sleep parameters, and that there would be an interaction effect between noise and noise sensitivity.

## Methods

### Participants

A total of 500 individuals aged 18 years and older who could understand and read Chinese were recruited. We excluded those who were deaf, needed hearing aids, had psychiatric illnesses, took pills or other medical treatments for sleep, were pregnant, had children under 2 years of age, were unwilling or unable to wear an ActiGraph GT9X Link, or were unwilling or unable to assess nocturnal noise exposure.

Sample size calculation was first based on the assessment of an estimated 30 factors associated with nonrestorative sleep or noise sensitivity and, using the usual rule of thumb of 10 participants per independent variable, a minimum of 300 participants were required. Second, to assess the moderating effect of noise sensitivity, we considered a conservative error of 0.1 on the standardized interaction effect, which, using a 95% confidence interval (CI), indicated a minimum sample size of 396. Hence, we planned to recruit 500 participants after considering the possibility of participants drops out. During recruitment, we approached 1625 individuals before 500 individuals consented to participate in this study. We excluded 584 individuals due to ineligibility. Moreover, 227 declined to participate and 314 were not at home more than 5 times during household visits.

### Sampling design and survey methodology

This was a population-based cross-sectional household survey conducted in Hong Kong from February 2018 to September 2019. The household sampling began by first obtaining a sample list based on the frame of quarters maintained by the Hong Kong Census and Statistics Department, which is the most complete, up-to-date, and authoritative sampling frame available in Hong Kong. Records of household addresses were organized in quarters and stratified by geographical districts and types of quarters. A systematic sampling design with fixed sampling intervals and non-repetitive random numbers was then applied to obtain a random sample of quarters. For the quarters selected, all households residing in the quarters were included in the survey.

Before household visits took place, notification letters outlying the study details, planned visit times, and interviewer identities were mailed to all targeted households. During the household visits, the eligibility criteria were assessed and written informed consent was obtained before taking study measurements.

After providing consent, a research assistant calibrated a noise dosimeter (Spark 706RC, Larson Davis Inc., US) or a sound level meter (NSRT Mk2, Convergence Instruments, Canada) using a CAL150 (Larson Davis Inc., US) at 94dBSPL and helped to identify an appropriate location for positioning the device. Each participant was then shown how to wear an ActiGraph GT9X Link (ActiGraph, US) strapped securely to the non-dominant wrist for a week and completed a battery of self-report questionnaires. After a week, another household visit was made to collect the devices and to distribute shopping coupons valued at HK$300 (around US$40) to each participant.

Ethics approval of this study was obtained from the Institutional Review Board of the University of Hong Kong/Hospital Authority Hong Kong West Cluster (Ref no.: UW17–011).

### Measurements

#### Objective measurements

##### Nocturnal noise exposure

Spark 706RC or the NSRT Mk2, both meeting American National Standards Institute (ANSI) S1.4, was used to record sound intensity levels for seven consecutive days to indicate nocturnal noise exposure [37]. It was placed on a stable surface within 2 m from where a participant slept and close to participant ear level during sleep and recorded A-weighted energy equivalence levels, set at 1-min intervals. Nocturnal noise exposure was calculated as the average equivalent continuous sound pressure level from 00:00 to 8:00 (*L*_*Aeq*, 8*h*_).

##### Actigraphy

Objective physiological sleep parameters, including sleep latency, sleep efficiency, time in bed (TIB), total sleep time (TST), wake after sleep onset (WASO), and awakenings and average awakenings, were measured by ActiGraph GT9X Link, and calculated by ActiLife (ActiGraph, US) using the Cole-Kriple algorithm, which has been validated among adults [38, 39]. The results of actigraphy have been demonstrated to be consistent with those of polysomnography [39]. Participants were required to wear for seven consecutive days, and a valid record required recording from at least four weekdays and one weekend day. Data obtained from the ActiGraph GT9X Link were corroborated by a sleep diary where participants recorded the time they went to bed and the time they woke up every day. Sleep latency was the duration between time in bed and the first minute that the algorithm scored “asleep” [38, 39]. TIB was calculated as the duration between time in bed and time out of bed, and TST refers to the total duration scored as “asleep” [38]. Sleep efficiency was the TST divided by the TIB [38]. WASO was the total time that the participants were awake after sleep onset and awakenings were the number of awakening episodes during the night, while average awakenings refers to the average duration of all awakening episodes [38].

#### Subjective measurements

##### Nonrestorative sleep scale (NRSS)

The Chinese version of the NRSS which includes four domains, namely, refreshment from sleep (e.g., Usually, do you think your sleep is restoring or refreshing?), the physical/medical symptoms of nonrestorative sleep (e.g., Do you feel that physical or medical problems are dragging you down), daytime functioning (e.g., What is your usual level of daytime energy?), and the affective symptoms of nonrestorative sleep (e.g., Do you feel depressed or down if you didn’t sleep well the night before?), has been demonstrated to be a valid and reliable instrument for measuring nonrestorative sleep [40]. The coefficient omega of the global score of the Chinese scale was 0.92 [40]. NRSS scores were standardized on a 0–100 scale, with higher scores indicating less nonrestorative sleep (i.e., better restorative sleep).

##### Weinstein noise sensitivity scale (WNSS)

The WNSS was developed to assess noise sensitivity. The traditional 18-item Chinese version of WNSS has been verified to be a reliable and valid scale to assess noise sensitivity [41]. Each item (e.g., I am easily awakened by noise) was rated on a 6-point Likert scale with a total score ranging from 18 to 108. This score was then standardized to a 0–100 scale. A higher total score indicates more sensitivity toward noise. The Chinese WNSS had a Cronbach’s alpha of 0.83 [41].

##### ENRICHD social support instrument (ESSI)

Social support was assessed via the ESSI. The Chinese version of ESSI consists of 6 items (e.g., Is there someone available to help with daily chores?), with each item rated on a 1–5 scale. The global score ranges from 6 to 30, with higher scores indicating a higher level of social support [42]. The internal consistency of the Chinese version scale is satisfactory (Cronbach’s alpha = 0.79) [42].

##### Patient health questionnaire (PHQ-15)

Psychosomatic symptoms were assessed using the Chinese version of the PHQ-15, which has been validated in a Chinese population [43]. It addresses 15 somatic symptoms (e.g., stomach pain), each assigned a score ranging from 0 (not bothered at all) to 2 (bothered a lot). The items cover the most prevalent DSM-IV somatization disorder somatic symptoms. The Chinese PHQ-15 had a Cronbach’s alpha of 0.79 [43].

##### Perceived stress scale (PSS)

The Chinese 10-item PSS was adopted to assess the level of stress in participants. The 10-item version has been demonstrated to have better validity and reliability than the 14-item and 4-item scales. Six of the ten items were positively worded (e.g., How often have you been upset because of something that happened unexpectedly”) and the other four items were negatively worded (e.g., How often have you felt confident about your ability to handle your personal problems?). Positive items were reversed-scored before computing the total score. Higher total scores indicate higher stress levels [44]. The Cronbach’s alpha of the Chinese PSS was reported as 0.85 [44].

##### Hospital anxiety and depression scale (HADS)

The Chinese version of the HADS comprises 14 items, seven of which measure anxiety (e.g., I feel tense or wound up) and seven measure depression (e.g., I still enjoy the things I used to enjoy). The subscales were scored independently, with each subscale score ranging between 0 and 21, with higher total scores indicating more severe anxiety/depressive symptoms [45]. Reliability, tested by Cronbach’s alpha, was 0.80 and 0.63 for the anxiety and depression subscales, respectively [45].

##### STOP-BANG questionnaire

The Chinese version of STOP-BANG questionnaire, which was modified from the STOP questionnaire, has been validated in Chinese adults in Hong Kong with high sensitivity, and is an appropriate screening instrument for obstructive sleep apnea (OSA) [46]. This scale addresses risk factors such as “BMI > 30kg/m^2^” and “Age > 50 years old”. Individuals are categorized as having a high risk of OSA when they score “Yes” on three or more items. It is a satisfactory questionnaire for OSA screening with a sensitivity up to 86% [46].

##### Sociodemographic and lifestyle characteristics

The sociodemographic and lifestyle data were self-reported in front of an interviewer. An investigator-developed self-reported information sheet which aimed to collect sociodemographic and lifestyle data from participants included age, sex (Male; Female), marital status (Single; Married/Cohabiting; Separated/Divorced/Widowed), education level (Primary school or below; Secondary; Bachelor or above), occupation (Working; Not working; Retired; Students), family income (from < HK$5000 to > HK$50000), exercise (from no to 5 h or more per week), smoking (Never; Quit; Yes), as well as consumption of alcohol (Never; Quit; Yes), cola, soda, coffee, and tea (Every day; Every week; Every month; Every year; Never). Season of conduction was summarized according to the recording time (Spring: Month 3–5; Summer: Month 6–8; Autumn: Month 9–11; Winter: Month 12–2). If the recording was conducted across different seasons, it was categorized according to the season with records for 4 days or more that were retained.

### Statistical analysis

First, we regressed nonrestorative sleep on each independent variable in a bivariate regression model. Then, a structured multiphase regression analysis was conducted to assess the association of nocturnal noise, noise sensitivity, and other variables with nonrestorative sleep and physiological sleep parameters. The structured multiphase regression model accounts for the possible causal relationship among the variables by first grouping them into sequential clusters [47]. Specifically, we defined five clusters such that variables in Cluster 1 could affect variables in Clusters 2, 3, 4 and 5, but not vice versa. Similarly, Cluster 2 variables may affect variables in Cluster 3, 4 and 5, but not vice versa, and so on. Cluster 1 included sociodemographic and time variables, i.e., age, sex, marital status, educational level, occupation, family income and season. Cluster 2 included lifestyle and living environment factors, i.e., exercise, smoking, alcohol, cola, soda, coffee, tea, social support, and nocturnal noise level. Cluster 3 included somatic symptoms, stress, anxiety, and depression levels. Cluster 4 included noise sensitivity. The physiological sleep parameters were included in Cluster 5. The structured multiphase regression analysis was then performed in five phases (i.e., enter regression). In Phase 1, a regression was conducted on all variables in Cluster 1. In Phase 2, a regression was conducted on all Cluster 2 variables adjusting for variables in Cluster 1. In Phase 3, variables in both Clusters 1 and 2 were considered as potential confounders for variables in Cluster 3, and so on. The effect of a variable in Cluster 1 was taken as that estimated in Phase 1, while the effect of a variable in Cluster 2 was taken as that estimated in Phase 2, and so on. Therefore, variables were not adjusted for the variables in the next cluster, which theoretically avoided potential over adjustment.

Moreover, all physiological sleep parameters were regressed on Clusters 1–4 separately. Furthermore, an interaction between noise sensitivity and nocturnal noise was also assessed based on each corresponding Phase 4 model.

All collected data were entered into SPSS version 23 (Armonk, NY: IBM Corp.) and cleaned by cross-checking with the original paper records. The significance level was set at 5%, and all estimates are accompanied by 95% CIs where appropriate. Regression was conducted with RStudio-1.2.1335. The adequacy of the regression models was assessed by examining the studentized residuals. The presence of multicollinearity was assessed by the variance inflation factor (VIF) with the “car” package under RStudio-1.2.1335 [48]. The VIF quantifies how much the variance is inflated by the existence of correlation among the independent variables in the model. A value of 1 indicated no correlation between independent variables while 1 < VIF ≤ 5 and > 5 indicated moderate correlation and high correlation, respectively [49].

## Results

### Social-demographic characteristics and nonrestorative sleep status of participants

The average age of the 500 participants was 39 years old (standard deviation [SD]: 12; range: 18–80). Table 1 shows the sociodemographic and lifestyle characteristics of the participants. All participants completed the 12-item NRSS without any missing values. The average nonrestorative sleep level was 64.77 (SD: 12.75) on a 0–100 scale. Table 2 shows the summary of nocturnal noise, noise sensitivity, nonrestorative sleep, physiological sleep variables, social support, somatic symptoms, stress, anxiety, and depression.

**Table 1:** Sociodemographic and lifestyle data for the 500 adults

Characteristics	Mean ± SD/n	Percent
Mean age ± SD	39 ± 12	
Sex		
Male	168	34%
Female	332	66%
Marital status		
Single	171	34.4%
Married/Cohabiting	307	61.2%
Separated/Divorced/Widowed	22	4.4%
Educational level (1 missing)		
Primary school or below	24	5%
Secondary	251	50%
Bachelor or above	224	45%
Occupation		
Working	370	74%
Not working	73	14%
Retired	15	3%
Students	42	8%
Family income (HK$, 10 missing)		
< 5000	10	2%
5000–9999	7	1.4%
10,000–14,999	17	3.4%
15,000–19,999	29	5.8%
20,000–24,999	46	9.2%
25,000–29,999	53	10.6%
30,000–34,999	68	13.6%
35,000–39,999	37	7.4%
40,000–44,999	55	11.0%
45,000–49,999	37	7.4%
> 50,000	131	26.2%
Season		
Spring	237	47.4%
Summer	141	28.2%
Autumn	51	10.2%
Winter	71	14.2%
Aerobic exercise per week		
No	170	34%
1 h	116	23%
2 h	88	18%
3 h	61	12%
4 h	28	6%
5 h or more	37	7%
Smoking		
Never	404	81%
Quit	38	7%
Yes	58	12%
Alcohol		
Never	270	54%
Quit	36	7%
Yes	194	39%
Cola		
Every day	15	3%
Every week	130	26%
Every month	170	34%
Every year	68	14%
Never	117	23%
Soda		
Every day	10	2%
Every week	78	16%
Every month	139	28%
Every year	57	11%
Never	216	43%
Coffee		
Every day	102	20%
Every week	104	21%
Every month	62	12%
Every year	19	4%
Never	213	43%
Tea		
Every day	108	22%
Every week	198	39%
Every month	81	16%
Every year	14	3%
Never	99	20%

*SD* standard deviation

**Table 2:** Summary of noise, noise sensitivity, sleep, social support, somatic symptoms, stress, anxiety and depression (*n* = 500)

	Mean	SD	Range	95%CI
**Nocturnal noise level (dBA)**	51.32	5.61	36.51, 70.67	50.82, 51.82
**WNSS (0–100)**	60.44	12.09	21.11, 94.44	59.38, 61.50
**NRSS (0–100)**	64.77	12.75	19.39, 100	63.65, 65.89
Refreshment from sleep	59.16	16.99	0, 100	57.67, 60.66
Physical/medical symptoms of NRS	68.60	18.39	21.88, 96.88	66.98, 70.22
Daytime functioning	63.01	15.47	0, 100	61.65, 64.37
Affective symptoms of NRS	65.65	21.30	7.69,100	63.78, 67.52
**Physiological sleep parameters**				
Latency (min)	7.13	6.95	0, 75.71	6.52, 7.74
Efficiency (%)	78.34	7.74	44.56, 97.84	77.66, 79.02
Time in bed (min)	441.10	65.17	199.29, 651.5	441.60, 444.09
Total sleep time (min)	345.07	57.38	178.43, 534.29	345.22, 345.84
Wake after sleep onset (min)	88.78	37.40	6.17, 245.4	85.50, 92.07
Awakenings (n)	25.84	7.91	4.29, 50.14	25.14, 26.53
Average awakenings (min)	3.48	1.33	1.17, 20.14	3.37, 3.60
**ESSI**	21.39	4.76	6, 30	20.98, 21.81
**PHQ**	3.93	3.88	0, 19	3.58, 4.30
**PSS**	15.60	5.31	0, 30	15.12, 16.10
**Anxiety**	4.43	3.47	0, 16	4.13, 4.75
**Depression**	5.15	3.44	0, 17	4.83, 5.48

*SD* standard deviation, *CI* confidence interval; *WNSS* Weinstein Noise Sensitivity Scale, *NRS* nonrestorative sleep, *NRSS* nonrestorative sleep scale, *ESSI* ENRICHD Social Support Instrument, *PHQ* physical health questionnaire, *PSS* perceived stress scale

### Spearman rank correlations between nocturnal noise, noise sensitivity, nonrestorative sleep, with physiological sleep parameters

Table 3 shows the associations between nocturnal noise, noise sensitivity, NRSS (four subscales), and the physiological sleep parameters. Nocturnal noise was significantly associated with only the physical/medical symptoms subscale of NRSS (r = 0.09, *p* < 0.05), TIB (r = − 0.15, *p* < 0.01) and TST (r = − 0.13, p < 0.01). Noise sensitivity was statistically significantly associated with NRSS (r = − 0.26, *p* < 0.01), as well as its four subscales, but not with any physiological sleep parameters.

**Table 3:** Spearman rank correlation between noise, noise sensitivity, nonrestorative sleep, and physiological sleep parameters (*n* = 500)

	Nocturnal noise	WNSS	NRSS	1	2	3	4	5	6	7	8	9	10	11
**Nocturnal noise**	1													
**WNSS**	−0.07	1												
**NRSS**	−0.05	−0.26^**^	1											
1. Refreshment from sleep	−0.04	− 0.29^**^	0.71^**^	1										
2. Physical/medical symptoms of NRS	−0.06	− 0.09^*^	0.77^**^	0.30^**^	1									
3. Daytime functioning	0.09^*^	−0.13^**^	0.66^**^	0.48^**^	0.27^**^	1								
4. Affective symptoms of NRS	0.05	− 0.27^**^	0.67^**^	0.33^**^	0.54^**^	0.26^**^	1							
**5. Latency**	− 0.03	− 0.05	− 0.08	− 0.09	−0.08	− 0.03	− 0.05	1						
**6. Efficiency**	−0.01	0.02	0.07	0.04	0.13^**^	−0.02	0.05	−0.34^**^	1					
**7. Time in bed**	−0.15^**^	0.01	0.06	0.16^**^	−0.03	0.10^*^	0.01	0.16^**^	−0.16^**^	1				
**8. Total sleep time**	−0.13^**^	0.01	0.09	0.15^**^	0.04	0.06	0.04	−0.07	0.44^**^	0.78^**^	1			
**9. Wake after sleep onset**	−0.04	0.02	−0.03	0.04	−0.10^*^	0.06	−0.05	0.22^**^	−0.91^**^	0.48^**^	−0.10^*^	1		
**10. Awakenings**	−0.09	0.06	−0.10^*^	−0.02	− 0.14^**^	0.04	− 0.12^**^	0.20^**^	− 0.58^**^	0.49^**^	0.11^*^	0.71^**^	1	
**11. Average awakenings**	0.01	−0.02	0.06	0.07	0.00	0.05	0.04	0.10^**^	−0.65^**^	0.16^**^	−0.25^*^	0.64^**^	−0.02	1

*WNSS* Weinstein Noise Sensitivity Scale, *NRSS* nonrestorative sleep scale, *NRS* nonrestorative sleep

^*^: *p*-value < 0.05; ^**^: *p*-value < 0.01

### Association between noise sensitivity, nocturnal noise and global scale of NRSS

Table 4 shows the results of bivariate regression of each independent variable on the global NRSS. Age, marital status, occupation, family income, season, exercise, consumption of cola and soda, social support, somatic symptoms, stress, depression, noise sensitivity, total sleep time and awakenings were associated with nonrestorative sleep.

**Table 4:** Linear regression of NRSS in Chinese adults^a^

Variable	Estimate	95% CI	***p***-value
Age	0.11	0.19, 0.20	0.017^*^
Sex (Ref: Male)			
Female	−1.55	− 3.92, 0.82	0.199
Marital status (Ref: Single)			
Married/Cohabiting	2.85	1.98, 6.71	< 0.001**
Separated/Divorced/Widowed	5.54	−0.07, 11.14	0.053
Educational level (Ref: Primary school or below)			
Secondary	− 1.97	−7.31, 3.36	0.468
Bachelor or above	−3.54	−8.91, 1.82	0.195
Occupation (Ref: Working)			
Not working	−1.55	−4.88, 1.79	0.363
Retired	−3.88	−9.35, 1.59	0.164
Students	−5.07	−9.13, −1.01	0.014^*^
Family income	0.53	0.12, 0.94	0.011^*^
Season (Ref: Spring)			
Summer	−3.93	−6.57, −1.29	0.004^**^
Autumn	−5.67	−9.49, −1.84	0.004^**^
Winter	−2.38	−5.73, 0.98	0.165
Exercise	1.15	0.44, 1.87	0.002^**^
Smoking (Ref: Never smoking)			
Quitted smoking	−2.40	−6.66, 1.85	0.267
Current smoking	−1.81	− 5.32, 1.71	0.314
Alcohol (Ref: Never alcohol)			
Quitted alcohol	−3.99	−8.43, 0.44	0.078
Current alcohol	−1.81	−4.16, 0.55	0.132
Cola	1.00	0.04, 1.95	0.040^*^
Soda	1.17	0.24, 2.09	0.013^*^
Coffee	0.45	−0.23, 1.13	0.197
Tea	−0.09	− 0.90, 0.72	0.823
Social support	0.82	0.59, 1.04	< 0.001^**^
Nocturnal Noise	−0.12	− 0.32, 0.08	0.231
Somatic symptoms	−1.88	−2.12, − 1.64	< 0.001^**^
Stress	−1.47	−1.63, − 1.30	< 0.001^**^
Anxiety	−2.10	− 2.37, − 1.84	0.055
Depression	− 1.80	− 2.09, − 1.51	< 0.001^**^
Noise sensitivity	− 0.27	− 0.36, − 0.18	< 0.001^**^
Latency	− 0.12	− 0.28, 0.04	0.147
Efficiency	0.09	− 0.05, 0.24	0.203
Time in bed	0.01	−0.00, 0.03	0.116
Total sleep time	0.02	0.00, 0.04	0.024^*^
Wake after sleep onset	−0.01	−0.04, 0.02	0.637
Awakenings	−0.14	−0.28, − 0.00	0.048^*^
Average Awakenings	0.58	−0.26, 1.43	0.174

^a^: All unadjusted results in the model, *NRSS* nonrestorative sleep scale, ^*^: *p*-value < 0.05; ^**^: *p*-value < 0.01

Table 5 shows the adjusted associations of the variables on the global NRSS. There were high explained variances for Phases 3 and 4, with values of 52.0 and 52.8%, respectively, and the VIF ranged from 1.03 to 1.56. People who had less family income, exercised less, had less social support, and had more somatic symptoms, stress, depression, and noise sensitivity, and in summer and autumn seasons, reported higher levels of nonrestorative sleep. Nocturnal noise level showed no significant association with the global score. However, one unit increase in noise sensitivity was associated with a − 0.08 (95% CI: − 0.15, − 0.01, *p* = 0.023) change in the global score. Furthermore, none of the physiological sleep parameters were associated with NRSS after adjusting for the above-mentioned variables while noise sensitivity remained associated with NRSS (− 0.08, 95% CI: − 0.15, − 0.01, *p* = 0.018). After excluding 36 individuals who were indicated as at high risk of OSA on the STOP-BANG, the relationship between noise sensitivity and global scale remained significant (− 0.08, 95% CI: − 0.15, − 0.01, *p* = 0.022).

**Table 5:** Structured multiphase regression of NRSS in Chinese adults (*n* = 489)

Variable	Estimate	95% CI	***p***-value
**Phase 1** (R^2^ = 6.8%, adjusted R^2^ = 4.3%)			
Age	0.01	−0.13, 0.12	0.912
Sex (Ref: Male)			
Female	−1.35	−3.85, 1.15	0.290
Marital status (Ref: Single)			
Married/Cohabiting	2.85	−0.10, 5.79	0.058
Separated/Divorced/Widowed	5.98	−0.37, 12.33	0.065
Educational level (Ref: Primary school or below)			
Secondary	−1.28	−6.60, 4.05	0.637
Bachelor or above	−2.75	−8.32, 2.83	0.334
Occupation (Ref: Working)			
Not working	−2.02	−5.73, 1.69	0.285
Retired	−4.60	−10.41, 1.22	0.121
Students	−1.82	−6.51, 2.88	0.447
Family income	0.52	0.07, 0.98	0.023^*^
Season (Ref: Spring)			
Summer	−2.91	−5.65, −0.17	0.038^*^
Autumn	−4.04	−8.01, − 0.07	0.046^*^
Winter	−0.95	−4.40, 2.49	0.587
**Phase 2** (R^2^ = 18.3%, adjusted R^2^ = 14.1%)			
Exercise	1.12	0.41, 1.82	0.002^**^
Smoking (Ref: Never smoking)			
Quitted smoking	−2.69	−6.88, 1.50	0.208
Current smoking	−2.47	−5.96, 1.03	0.166
Alcohol (Ref: Never alcohol)			
Quitted alcohol	−3.25	−7.64, 1.14	0.146
Current alcohol	−1.49	−3.90, 0.91	0.223
Cola	0.17	−0.91, 1.25	0.761
Soda	0.46	−0.54, 1.46	0.362
Coffee	0.36	−0.31, 1.02	0.289
Tea	0.14	−0.67, 0.95	0.736
Social support	0.73	0.50, 0.96	< 0.001^**^
Nocturnal Noise	−0.04	−0.24, 0.17	0.736
**Phase 3** (R^2^ = 54.5%, adjusted R^2^ = 51.7%)			
Somatic symptoms	−0.95	−1.21, − 0.69	< 0.001^**^
Stress	− 0.73	− 0.94, − 0.52	< 0.001^**^
Anxiety	− 0.35	− 0.70, 0.01	0.055
Depression	− 0.61	− 0.90, − 0.31	< 0.001^**^
**Phase 4** (R^2^ = 55.0%, adjusted R^2^ = 52.2%)			
Noise sensitivity	−0.08	− 0.15, − 0.01	0.023^*^
**Phase 5** (R^2^ = 56.1%, adjusted R^2^ = 52.6%)			
Latency	−0.15	− 0.47, 0.17	0.359
Efficiency	−0.20	−0.85, 0.46	0.555
Time in bed	−0.01	−0.32, 0.31	0.975
Total sleep time	0.02	−0.30, 0.35	0.890
Wake after sleep onset	−0.03	−0.31, 0.26	0.826
Awakenings	−0.07	−0.27, 0.14	0.518
Average Awakenings	0.62	−0.31, 1.56	0.192

*NRSS* nonrestorative sleep scale, *CI* confidence interval

^*^: *p*-value < 0.05; ^**^: *p*-value < 0.01

### Association between noise sensitivity, nocturnal noise and subscales of NRSS

Analyses were conducted on the four NRSS subscales. Nocturnal noise level was only associated with affective symptoms of nonrestorative sleep scale of the NRSS (b = 0.37, 95% CI: 0.02, 0.72, *p* = 0.040). For every unit increase in noise sensitivity, there was a − 0.24 (95% CI: − 0.36, − 0.12; *p* < 0.001) and − 0.22 (95% CI: − 0.36, − 0.08; *p* = 0.002) change in the refreshment from sleep and affective symptoms of nonrestorative sleep scales, respectively, after adjusting for the above-mentioned confounders.

### Association between noise sensitivity, nocturnal noise and physiological sleep parameters

Table 6 shows the associations between physiological sleep parameters with nocturnal noise and noise sensitivity after adjusting for sociodemographic and lifestyle characteristics, social support, somatic symptoms, stress, anxiety, and depression. The VIF ranged from 1.03 to 1.56. Nocturnal noise reduced TIB (b = − 1.65, 95% CI: − 2.77, − 0.52, *p* = 0.004), TST (b = − 1.61, 95% CI: − 2.59, − 0.62, *p* = 0.001), and awakenings (b = − 0.16, 95% CI: − 0.30, − 0.03, *p* = 0.018). However, noise sensitivity did not show any significant changes with physiological sleep parameters.

**Table 6:** Effects of nocturnal noise and noise sensitivity on physiological sleep parameters (n = 489)

Nocturnal noise				WNSS		
Variables	Estimate	95% CI	***p-value***	Estimate	95% CI	***p-value***
Latency	−0.01	(− 0.12, 0.11)	0.915	− 0.05	(− 0.07, 0.05)	0.083
Efficiency	−0.06	(− 0.19, 0.07)	0.365	− 0.01	(− 0.07, 0.05)	0.855
Time in bed	−1.65	(−2.77, − 0.52)	0.004^**^	0.10	(−0.40, 0.61)	0.692
Total sleep time	−1.61	(−2.59, −0.62)	0.001^**^	0.05	(−0.39, 0.49)	0.830
Wake after sleep onset	−0.01	(−0.65, 0.63)	0.975	0.10	(−0.19, 0.39)	0.500
Awakenings	−0.16	(−0.30, − 0.03)	0.018^*^	0.04	(− 0.02, 0.10)	0.202
Average Awakenings	0.02	(−0.01, 0.04)	0.189	0.00	(−0.01, 0.01)	0.819

*CI* confidence interval, *WNSS* Weinstein Noise Sensitivity Scale

^*^: *p*-*value* < 0.05; ^**^: *p*-*value* < 0.01

### Moderation of noise sensitivity on the relationship between nocturnal noise and sleep

For both nonrestorative sleep and physiological sleep parameters, there was no significant interaction between nocturnal noise and noise sensitivity (*p* ≥ 0.104).

## Discussion

This was the first study to assess the association between nocturnal noise, noise sensitivity, and nonrestorative sleep using standardized scales and adjusting for relevant confounders. The results revealed that noise sensitivity, as well as environment, lifestyle, physical and psychosocial health were associated with nonrestorative sleep.

The average nonrestorative sleep level of Chinese adults in Hong Kong was 64.77 (SD: 12.75) on the 0–100 scale, which indicated that the degree of feeling refreshed after sleep was only around 65%. This is lower than that (80.90 on the 0–100 scale) in the general Canadian population, indicating Hong Kong adults had a higher level of nonrestorative sleep [50]. Indeed, a previous study indicated that 39.4–68.6% of Hong Kong adults had insomnia [51, 52], which was higher than that of Canadians [53]. Therefore, sleep conditions may be generally poor in Hong Kong adults. Problems such as nonrestorative sleep deserve more attention and interventions that target reductions in nonrestorative sleep would be desirable.

The average nocturnal noise level in the participants’ bedrooms was 51.32 dBA (SD: 5.61), which was above the suggested level of 30–40dBA at night [37]. This suggests an increased risk of sleep disturbances and medical conditions, and poor well-being [37]. Moreover, when the nocturnal noise level is greater than 50dBA, the risk of hypertension and myocardial infarction is also increased [37]. Nonrestorative sleep was independent of nocturnal noise level while TIB and TST were shown to be negatively associated with noise in this study. This is consistent with a previous study that revealed the relationships of noise, noise annoyance, and objective and subjective sleep parameters [54]. People who live in noisy areas may be more easily influenced by noise, and tend to have less TIB [55]. Furthermore, noise has been shown to fragment sleep, reduce sleep continuity and TST, and increase awakenings and shifts between stages of sleep [56]. These factors may result in less “asleep” time for individuals. However, the number of awakenings was negatively associated with nocturnal noise in this study which conflicts with previous studies [56]. Notwithstanding, the bivariate correlation demonstrated that the number of awakenings was not associated with noise. This might be due to people having more awakenings in autumn in this study. It might also be owing to awakenings being more associated with noise events, maximum noise level or subjective perceptions such as tolerance of noise or habituation to noise [57, 58]. This might also explain why no association between the objective measures of sound and latency and WASO was found in this study. Furthermore, despite the number of awakenings in our study being comparable to a previous study [59], actigraphy, which detected awakenings based on movements, was found to note more awakenings compared with video observation [60]. Nevertheless, as nocturnal noise was associated with physiological sleep parameters, a policy of restricting nocturnal noise would be desirable.

Consistent with our hypothesis, there was an association between noise sensitivity and nonrestorative sleep. However, noise sensitivity was not associated with physiological sleep parameters after adjusting for nocturnal noise, sociodemographic characteristics, lifestyle factors, physical and psychosocial health. It is unlikely that people who are more noise sensitive would have substantially more emotional responses to noise when they are asleep, as they should then also have more changes in their physiological sleep levels under the general stress model. Alternatively, the potential influence of noise sensitivity on nonrestorative sleep may be attributed to the higher vulnerability of noise-sensitive people to daytime noise exposure whose stress-related responses have not recovered by the time they go to sleep. Indeed, prolonged daytime noise exposure has been reported to be associated with reduced nighttime slow wave sleep (SWS) and sleep efficiency, which may then result in elevated cortisol and autonomic nervous system activity [61]. Therefore, people who are sensitive to noise would have higher stress-related responses that impacted nonrestorative sleep. However, in our random sample study, it was not feasible to measure actual daytime noise exposure. Well-designed studies with fewer subjects that incorporate daytime noise measurements will be necessary to confirm this hypothesis. In addition to the global scale of NRSS, higher noise sensitivity was also associated with less refreshment from sleep and more affective symptoms. The refreshment from sleep scale shares a similar interpretation as the global scale. For affective symptoms, they have been shown to be influenced by introversion and extroversion [62], which are two personality traits that have also been shown to be associated with noise sensitivity [23].

The additional consideration of the somatic symptoms, stress, and depression in Phase 3 added much more variance compared with the variables considered in Phase 2, indicating the importance of such variables in accounting for the variation of nonrestorative sleep. Currently, people are exposed to various psychosocial risk factors [63]. The association between nonrestorative sleep with stress and depression was consistent with a previous study [30]. Stressful events and psychosocial stressors could impact sleep by increasing the activity of the hypothalamic–pituitary–adrenal axis and release of cortisol while decreasing the production of melatonin [18], reflected by increased sleep latency and awakenings, and reduced SWS and sleep efficiency [64, 65]. Stress could also induce depressive feelings [66] while those depressive individuals were characterized by a decrease of SWS and sleep efficiency in addition to an increase of the percentage and density of rapid-eye-movement (REM) [67]. In addition, nonrestorative sleep was found to be associated with chronic medical disorders such as fibromyalgia syndrome [68]. Individuals who suffer from chronic somatic symptoms like pain may have difficulty falling asleep and more awakenings after sleep onset, which can then increase the risk of poor sleep and nonrestorative sleep [68]. Moreover, pain can form a vicious cycle with stress and depression which then exacerbate nonrestorative sleep [69]. Conclusively, the interaction between physical and psychological health increased the likelihood of nonrestorative sleep.

Individuals who had higher family income, more exercise and social support had less nonrestorative sleep in this study. Higher-level family income is associated with less stress and mental disorders [70]. Regular exercise and adequate social support are not only stress relievers but also can strengthen individual resilience to stress [32, 71, 72]. Therefore, we anticipated that these factors were able to reduce stress and thereby decrease nonrestorative sleep levels. Moreover, the association between nonrestorative sleep and exercise was consistent with a previous study which indicated that people who had regular exercise reported less nonrestorative sleep [31]. Higher intensity exercise was associated with shorter sleep latency and fewer awakenings [73]. Exercise could also shorten the N1 stage of non-rapid-eye-movement sleep, which is the stage of very light sleep, while increase REM sleep, sleep continuity, and sleep efficiency [74]. Therefore, exercise could be beneficial for relieving nonrestorative sleep.

In summer and autumn, people had higher levels of nonrestorative sleep compared with spring in this study. This might be attributed to the physical environment being less comfortable, as the temperature and humidity in Hong Kong in summer and autumn are high. A previous study showed that too stuffy bedrooms were associated with a higher likelihood of nonrestorative sleep [30].

Lastly, this study indicated that none of the obtained physiological sleep parameters were associated with nonrestorative sleep in the adjusted model. Researchers have proposed a possible association between alpha activity and nonrestorative sleep among people with chronic fatigue syndrome. However, the fact that people without such symptoms also had nonrestorative sleep cast doubt on this link [1]. As there remains a lack of study on the relationship between objective physiological sleep parameters and nonrestorative sleep, a more precise and functional device that can reveal more sleep parameters is worthy of research—to test the relationship between specific physiological sleep parameters and nonrestorative sleep.

This study adds to the literature that noise sensitivity is associated with nonrestorative sleep, which informs the need to assess noise sensitivity in individuals experiencing nonrestorative sleep, as well as developing interventions to reduce noise sensitivity. In addition, this study also adds that the degree of feeling refreshed after sleep was only around 65% in the Chinese population. Interventions that target for reducing nonrestorative would be desirable. Despite our efforts to explore potential factors associated with nonrestorative sleep, there are several study limitations that deserve attention. Due to the cross-sectional study design, we were unable to identify causative links between nonrestorative sleep and noise sensitivity. As in other self-reported surveys, there might be the problem of common method variance that biased the derived associations. Common method variance may arise when responses from a subject are driven by factors other than the underlying constructs, such as the tendency for consistency, unwillingness to give true responses, and limited ability to respond [75]. However, we have used well-tested instruments and the survey was conducted in a household setting when the study subjects were completing the survey in front of an interviewer. Thus, the potential impact of common method variance should be minimal. Nevertheless, longitudinal studies are desirable to verify the associations. Furthermore, the mechanism underlying why noise-sensitive people have higher nonrestorative sleep is worth studying since people are considered to be less reactive to the outside environment when sleeping. Second, despite controlling a number of covariates, noise sensitivity usually co-exists with some other environmental sensitivities [24], noise sensitivity might indicate a general predisposition of individuals to environmental stressors. Therefore, the observed association between noise sensitivity and nonrestorative sleep might also be attributed to such environmental sensitivities or susceptibility to stressors. Hence, a further study that covers more environmental sensitivities and stressors is desirable to disclose such potential relationships. Third, we had taken measurements of daily objective sleep quality and nocturnal noise for around a week, further analysis incorporating these as outcomes may consider using mixed effects model or generalized estimating equations that account for the extra covariance among the repeated measurements. Fourth, daytime noise, which may also influence nonrestorative sleep and physiological sleep parameters, was not investigated in this study. This would require the assessment of personal noise exposure by asking the participants to carry a noise dosimeter all day. However, this can be highly demanding to the participants and innovations in assessing personal noise exposure would be helpful in this regard. Moreover, other noise characteristics such as maximum noise level, number of noise events and vibrations are known to be associated with sleep disturbances. Therefore, a future study that including such variables is desirable to investigate the factors associated with physiological sleep parameters and nonrestorative sleep. Lastly, a structural equation model approach deserves consideration for identifying a more structured model with possible modifiers and mediators.

## Conclusions

In conclusion, noise sensitivity was associated with nonrestorative sleep, and nocturnal noise level was associated with physiological sleep parameters. Furthermore, family income, season, exercise, social support, somatic symptoms, stress, and depression were also related with nonrestorative sleep. Interventions targeting to reduce noise sensitivity or the levels of other risk factors while increasing levels of protective factors are desirable and need to be developed. In addition, the policy of restricting nocturnal noise level is necessary for improving objective sleep conditions for residents of highly urbanized areas such as Hong Kong.

## Data Availability

The datasets generated and/or analyzed during the current study are not publicly available due to the restrictions of the local ethics committee and institutional data security and privacy policies. The data access request needs institutional and ethics committee’s approval.

## Abbreviations

CI: Confidence interval
ESSI: ENRICHD Social Support Instrument
HADS: Hospital Anxiety and Depression Scale
NRS: Nonrestorative sleep
NRSS: Nonrestorative sleep scale
OSA: Obstructive sleep apnea
PHQ: Patient health questionnaire
PSS: Perceived stress scale
REM: Rapid-eye-movement
SD: Standard deviation
SWS: Slow wave sleep
TIB: Time in bed
TST: Total sleep time
VIF: Variance inflation factor
WASO: Wake after sleep onset
WNSS: Weinstein Noise Sensitivity Scale
